# Association of tumor markers CA 15-3, CEA, and CA 125 with [^18^F]NaF PET findings in breast cancer patients

**DOI:** 10.3389/fonc.2025.1673504

**Published:** 2025-11-21

**Authors:** Arvin Naeimi, Sara Harsini, Vilma Derbekyan, Gad Abikhzer, Marc Hickeson, Shawn Karls, Farzad Abbaspour

**Affiliations:** 1 Department of Radiology, McGill University Health Center, McGill University, Montreal, QC, Canada; 2 Student Research Committee, School of Medicine, Guilan University of Medical Sciences, Rasht, Iran; 3 Department of Molecular Imaging and Therapy, BC Cancer, Vancouver, BC, Canada; 4 Department of Medical Imaging, University of Toronto, Toronto, ON, Canada; 5 Department of Medical Imaging, Division of Nuclear Medicine, McGill University Health Center, McGill University, Montreal, QC, Canada; 6 Department of Medical Imaging, Division of Nuclear Medicine, Jewish General Hospital, McGill University, Montreal, QC, Canada; 7 Division of Nuclear Medicine, Department of Medicine, The Ottawa Hospital and University of Ottawa, Ottawa, ON, Canada

**Keywords:** PET, ^18^F-NaF, breast cancer, CA 15-3, CEA, CA 125, bone metastases, tumor marker

## Abstract

**Introduction:**

Breast cancer often metastasizes to bone, and [^18^F]NaF PET is commonly used to detect skeletal involvement. This study examines the association of serum CA 15-3, CEA, and CA 125 with [^18^F]NaF PET findings in breast cancer to guide clinical decision-making.

**Methods:**

This retrospective study included 360 breast cancer patients who underwent [^18^F]NaF PET. Associations between serum tumor markers (CA 15-3, CEA, CA 125) and [^18^F]NaF PET findings and lesion count were analyzed. Optimal cut-off values for predicting [^18^F]NaF PET positivity were determined using ROC analysis. Multivariable logistic regression identified independent predictors.

**Results:**

Among 360 patients (mean age 61.1 ± 13.9 years), median serum CA 15-3, CEA, and CA 125 levels were significantly elevated in patients with positive versus negative PET scans (all p<0.001). Marker levels revealed a dose–response relationship, rising with increasing numbers of skeletal lesions. In multivariable analysis, CA 15-3 (OR 1.053, p=0.002) and CEA (OR 1.264, p=0.001) independently predicted PET positivity, whereas CA 125 showed a marginal trend (p=0.081). ROC analysis identified optimal cut-offs of 19.25 U/mL for CA 15-3 (sensitivity 70.1%, specificity 90.4%, AUC 0.837) and 3.15 ng/mL for CEA (sensitivity 65.6%, specificity 85.2%, AUC 0.821). Combined model incorporating all three markers (probability cut-off 0.29) improved diagnostic performance (AUC 0.863; sensitivity 79.7%, specificity 92.3%). Invasive lobular histology and restaging indication were significant predictors of PET positivity.

**Conclusion:**

Elevated CA 15–3 and CEA independently predict [^18^F]NaF PET positivity in breast cancer. Optimal cut-offs were 19.25 U/mL for CA 15-3 (sensitivity 70.1%, specificity 90.4%, LR + 7.38, AUC 0.837) and 3.15 ng/mL for CEA (sensitivity 65.6%, specificity 85.2%, LR + 4.43, AUC 0.821). The clinical utility of CA 15–3 and CEA lies in rule-in and risk-stratification strategies. Patients above these thresholds, particularly those with invasive lobular carcinoma undergoing restaging, may benefit from prioritized [^18^F]NaF PET evaluation or improved interpretation of equivocal PET findings. CA 15–3 threshold, lower than routine laboratory reference, may guide aggressive screening and prioritized [^18^F]NaF PET in patients with high clinical suspicion. Multivariable model combining CA 15-3, CEA, and CA 125 (probability cut-off 0.29) improved diagnostic performance (sensitivity 79.7%, specificity 92.3%, AUC 0.863). Integrating CA 15–3 and CEA into clinical decision-making may enable a nuanced, risk-adapted approach, optimizing metastasis detection and resource allocation.

## 1 Introduction

Breast cancer is the most common malignancy and the second leading cause of cancer-related deaths among women globally. In 2024, an estimated 297,790 new cases of breast cancer are expected to be diagnosed in the United States, with 43,170 resultant deaths​​ (1). Early diagnosis and continuous monitoring are crucial for the effective management of breast cancer (2). Various imaging techniques, such as mammography, ultrasound, and magnetic resonance imaging (MRI), are commonly used for breast cancer screening (3). Furthermore, advanced imaging modalities, including positron emission tomography (PET) and computed tomography (CT), are integral in diagnosing and monitoring breast cancer at various stages​​ (2).

Despite advancements in imaging technologies, breast cancer continues to be a leading cause of mortality, primarily due to its high propensity for metastasis, particularly to bones (4–6). Bone metastasis is a significant concern, with over half of patients with recurrent breast cancer developing metastases in the skeleton, primarily affecting the skull, ribs, spine, and pelvis (7). These metastases lead to severe skeletal complications such as pathological fractures, hypercalcemia, nerve compression, and extreme pain, significantly increasing morbidity and mortality (8, 9). Hence, early detection and timely treatment of bone metastases are essential to improve patient outcomes.

Traditionally, bone scintigraphy using technetium-99m–labeled diphosphonates (e.g., [^99m^Tc]Tc-MDP, [^99m^Tc]Tc-HDP, and [^99m^Tc]Tc-DPD) has been widely employed for detecting bone metastases, but these tracers share limitations in sensitivity and specificity (10, 11). Recent advancements have demonstrated the superiority of combining multiple imaging techniques for better evaluation of skeletal metastases in breast cancer (12). Specifically, sodium [^18^F]fluoride ([^18^F]NaF) PET/CT has emerged as a more accurate alternative, offering higher spatial and contrast resolution, shorter scanning times, and comprehensive whole-body imaging (13, 14). [^18^F]NaF PET visualizes osteoblastic activity with greater accuracy than [^99m^Tc]Tc-MDP bone scans, facilitating better detection of bone metastases (15).

Typically, the diagnosis of bone involvement relies on clinical signs or symptoms, which may delay the detection of metastases (16). Incorporating adjunctive serum biomarkers could enhance early diagnosis. Among various biomarkers, cancer antigen 15-3 (CA 15-3), carcinoembryonic antigen (CEA), and cancer antigen 125 (CA 125) have shown promise as diagnostic and prognostic indicators in breast cancer (17). These biomarkers reflect tumor burden and, when used alongside imaging techniques like PET/CT, may facilitate earlier detection of metastatic disease and improve patient management (18, 19).

This study investigates the correlation between [^18^F]NaF PET results and serum levels of CA 15-3, CEA, and CA 125 in breast cancer patients. Understanding this association aims to determine the optimal timing for [^18^F]NaF PET scans, potentially enhancing early detection and management of bone metastases in breast cancer patients.

## 2 Methods

### 2.1 Study design and participants

The database at McGill University Health Centre was retrospectively reviewed to select patients with breast cancer who underwent [^18^F]NaF PET at our institution between May 2016 and December 2017. We included patients with (1) a histopathologically confirmed diagnosis of breast cancer, (2) a high likelihood of bone metastases based on clinical stage (e.g., locally advanced breast cancer), signs (e.g., hypercalcemia, elevated alkaline phosphatase) or symptoms (e.g., bone pain), and (3) a maximum interval of three months between the PET study and the CA 15-3, CEA, CA 125, ALP, and LDH measurements. This cohort represents all eligible patients identified in the electronic medical records. Patient demographics, relevant oncologic history, laboratory values, and tumor pathology data were recorded. Tumor marker measurements were performed using Beckman Coulter assays. This study was approved by the Research Ethics Board (REB) of the McGill University Health Centre (MUHC; REB number: 2025-10862) and conducted in accordance with the Declaration of Helsinki. The requirement for informed consent was waived by the MUHC REB due to the retrospective nature of the study.

### 2.2 Procedures

An intravenous dose of [^18^F]NaF, ranging from 185 to 370 MBq (5–10 mCi), was administered. Patients were asked to void immediately before scanning. Image acquisition began at a minimum of 60 minutes after injection using a GE Discovery ST scanner. Total-body PET images (from vertex to toes) were obtained with the patient’s arms positioned at their sides. The average acquisition time per bed position was 1.5 minutes. The PET images were reconstructed using a standard iterative algorithm with a minimum matrix size of 128 × 128 for PET. PET data were analyzed in three orthogonal planes.

### 2.3 Image interpretation

Experienced nuclear medicine physicians interpreted the images using an Xeleris IV workstation (GE Healthcare). They completed a qualitative interpretation case report form, documenting the number of positive lesions (0, 1, 2, 3, 4, 5, or >5). With access to all clinical data, the physicians categorized the scans as positive, equivocal, or negative based on their diagnostic confidence. Scans were classified as positive if they showed focal and intense tracer uptake that was not associated with benign processes. Tracer uptake related solely to benign conditions, such as degenerative changes, enthesopathy, or iatrogenic and post-traumatic lesions, led to the scan being considered negative. Scans were deemed equivocal when tracer uptake was neither focal nor intense and could not be definitively linked to a benign cause. To confirm the initial interpretation, particularly for equivocal scans, follow-up within six months was reviewed using CT, MRI, follow-up [^18^F]NaF PET, [^18^F]FDG PET, MDP bone scan, biopsy, or clinical data, where available.

### 2.4 Statistical analysis

Continuous variables were summarized as mean ± standard deviation (SD) or median with interquartile range (IQR), and categorical variables as counts and percentages. Normality was assessed using the Kolmogorov–Smirnov test. Differences in tumor marker levels (CA 15-3, CEA, CA 125) across [^18^F]NaF PET result categories and lesion-count groups were evaluated using Kruskal–Wallis tests, with *post-hoc* pairwise comparisons performed using Mann–Whitney U tests. Bonferroni correction was applied to comparisons between PET result categories. Dose–response relationships between lesion count and tumor marker levels were examined using linear regression for log-transformed CA 15–3 and CEA, and Spearman rank correlation for CA 125. Locally weighted scatterplot smoothing (LOESS) curves were overlaid to visualize trends. Associations with [^18^F]NaF PET positivity were assessed using multivariable logistic regression. Two exploratory enter models were constructed: a biomarker model (age, CA 15-3, CEA, CA 125, ALP, and LDH) and a clinicopathological model (receptor status, histology, tumor grade, TNM staging, and indication), with all variables entered simultaneously to estimate adjusted associations. Diagnostic performance of tumor markers was assessed using receiver operating characteristic (ROC) curve analysis. Optimal cut-off values were determined using Youden’s index, and sensitivity, specificity, positive predictive value (PPV), negative predictive value (NPV), likelihood ratios (LR), and the area under the curve (AUC) were calculated. To develop a multivariable predictive model, stepwise logistic regression was applied to a pool of candidate variables, including age, CA 15-3, CEA, CA 125, ALP, and LDH. The final model retained CA 15-3, CEA, and CA 125, whereas age, ALP, and LDH were not retained. Predicted probabilities from this model were used to generate an ROC curve, and the optimal probability threshold was determined using the Youden index (0.29). Diagnostic performance metrics at this threshold (sensitivity, specificity, PPV, NPV, LR+, LR–, and AUC) were calculated and compared with those of individual tumor markers. All analyses were performed using SPSS version 25 (IBM Corp., Armonk, NY, USA) and Stata version 17 (StataCorp, College Station, TX, USA), with the significance level set at 0.05.

## 3 Results

### 3.1 Patient characteristics

Our search of medical records identified 360 female patients (mean age 61.1 ± 13.9 years) who met the inclusion criteria. Most patients had invasive ductal carcinoma (62.2%). A total of 119 patients (33.1%) were evaluated for staging, and 241 patients (66.9%) for restaging. Patient characteristics are summarized in **Table 1**. Overall, the cohort had median baseline serum levels of 13.6 U/mL for CA 15-3 (IQR 8.7–21.8), 1.9 ng/mL for CEA (IQR 1.1–3.9), 12.0 U/mL for CA 125 (IQR 8.0–24.5), 68.5 U/L for ALP (IQR 54.3–84.8), and 170 U/L for LDH (IQR 151–197). Laboratory assessments were conducted on average 25.2 ± 29.1 days relative to [^18^F]NaF PET imaging.

**Table 1:** Baseline characteristics of participants.

Characteristic	N=360
Age (y)^£^	61.1 (13.9)
Female sex^§^	360 (100%)
Height (cm)^£^	163.5 (8.1)
Weight (kg)^£^	76.2 (15.8)
Baseline lab parameters	
CA 15-3 (U/mL)^‡^	13.55 (8.73–21.78)
CEA (ng/mL)^‡^	1.90 (1.10–3.90)
CA 125 (U/mL)^‡^	12.00 (8.00–24.50)
ALP (U/L)^‡^	68.50 (54.25–84.75)
LDH (U/L)^‡^	170.00 (151.00–197.00)
T classification^§^	
T0	9 (2.5%)
T1	91 (25.3%)
T2	95 (26.4%)
T3	38 (10.6%)
T4	12 (3.3%)
Unknown	115 (31.9%)
N classification^§^	
N0	98 (27.2%)
N1	101 (28.0%)
N2	20 (5.6%)
N3	18 (5.0%)
Unknown	123 (34.2%)
M classification^§^	
M0	193 (53.6%)
M1	38 (10.6%)
Unknown	129 (35.8%)
Tumor histology^§^	
Invasive ductal carcinoma	224 (62.2%)
Invasive lobular carcinoma	28 (7.8%)
Mixed	4 (1.1%)
Unknown	104 (28.9%)
Tumor grade^§^	
Well-differentiated	25 (6.9%)
Moderately-differentiated	134 (37.2%)
Poorly-differentiated	76 (21.1%)
Unknown	125 (34.7%)
ER status^§^	
Positive	201 (55.8%)
Negative	49 (13.6%)
Unknown	110 (30.6%)
PR status^§^	
Positive	170 (47.2%)
Negative	80 (22.2%)
Unknown	110 (30.6%)
HER-2 status^§^	
Positive	52 (14.4%)
Negative	186 (51.7%)
Unknown	122 (33.9%)
Indication^§^	
Staging	119 (33.1%)
Restaging	241 (66.9%)
Final PET result	
Negative	240 (66.7%)
Equivocal	29 (8.1%)
Positive	91 (25.3%)
Number of lesions	
0	240 (66.7%)
1	20 (5.6%)
2	15 (4.2%)
3	10 (2.8%)
4	9 (2.5%)
5	7 (1.9%)
>5	59 (16.4%)

^£^Mean (standard deviation); ^‡^Median (interquartile range); ^§^Number (%); CA 15-3, cancer antigen 15-3; CEA, carcinoembryonic antigen; CA 125, cancer antigen 125; ALP, alkaline phosphatase; LDH, lactate dehydrogenase; ER, estrogen receptor; PR, progesterone receptor; HER-2, Human epidermal growth factor receptor-2. P-value < 0.05 is statistically significant.

After follow-up, 10 of the 29 initially equivocal PET cases were confirmed as true positive and 19 as true negative. Within the equivocal group, serum tumor marker levels (CA 15-3, CEA, and CA 125) did not differ significantly between patients ultimately classified as true positive versus true negative (all p > 0.05). Specifically, CA 15–3 was slightly higher in true positive cases (median 15.3 U/mL, IQR 10.9–18.2) compared with true negative cases (median 12.1 U/mL, IQR 9.5–14.7; Mann–Whitney U = 45.0, p = 0.33). CEA values, which were normally distributed in this subgroup, were nearly identical (2.46 ± 1.72 vs. 2.46 ± 1.64 ng/mL; U = 18.0, p = 0.77), and CA 125 levels were comparable (median 19.0 U/mL, IQR 7.0–32.6 vs. 24.0 U/mL, IQR 18.0–30.0; U = 25.0, p = 0.22) between true positive and true negative cases within the equivocal group.

In the overall cohort, one patient initially classified as [^18^F]NaF-positive was ultimately confirmed true negative, while three initially [^18^F]NaF-negative patients were later confirmed true positive.

### 3.2 [^18^F]NaF PET results and tumor marker associations

[^18^F]NaF PET results were negative in 240 patients (66.7%), positive in 91 (25.3%), and equivocal in 29 (8.1%) (**Table 1**). Median levels of CA 15-3, CEA, and CA 125 differed significantly across PET result categories (P < 0.001 for CA 15–3 and CEA; P = 0.001 for CA 125; **Table 2**). Median CA 15–3 values increased from 11.4 U/mL (IQR 7.9–15.2) in the negative group to 12.3 U/mL (IQR 8.3–17.5) in the equivocal group and 32.6 U/mL (IQR 15.9–77.6) in the positive group (χ² = 48.89, P < 0.001); *post-hoc* pairwise comparisons with Bonferroni correction showed significantly higher levels in positive versus negative and positive versus equivocal groups (P < 0.001 for both), whereas negative versus equivocal differences were not significant (P = 0.722). CEA exhibited a similar pattern, with medians of 1.5 ng/mL (IQR 0.95–2.5), 2.3 ng/mL (IQR 0.9–3.95), and 7.3 ng/mL (IQR 2.3–29.7) across negative, equivocal, and positive groups (χ² = 45.77, P < 0.001); positive cases had significantly higher levels than negative (P < 0.001, Bonferroni-adjusted) and equivocal (P = 0.011, Bonferroni-adjusted) groups, while negative versus equivocal differences were not significant (P = 0.701, Bonferroni-adjusted). For CA 125, median values were 10.0 U/mL (IQR 7.0–19.5), 21.0 U/mL (IQR 8.0–29.0), and 32.6 U/mL (IQR 16.0–55.0) in negative, equivocal, and positive groups, respectively (χ² = 13.43, P = 0.001); *post-hoc* comparisons with Bonferroni adjustment indicated significantly higher levels in positive versus negative (P < 0.001) and non-significant differences involving the equivocal group (positive vs equivocal P = 1; negative vs equivocal P = 0.319).

**Table 2:** CA 15-3, CEA, and CA 125 tumor marker levels according to [^18^F]NaF PET results.

Marker	Negative [^18^F]NaF PET median (IQR)	Positive [^18^F]NaF PET median (IQR)	Suspicious [^18^F]NaF PET median (IQR)	Kruskal–Wallis χ² (df = 2)	Overall p-value	Pairwise comparisons (Bonferroni-adjusted)
CA 15-3	11.4 (7.9–15.2)	32.6 (15.9–77.6)	12.3 (8.3–17.5)	48.89	**<0.001**	Pos > Neg (**p < 0.001**); Pos > Susp (**p < 0.001**); Neg vs Susp ns (p = 0.722)
CEA	1.5 (0.95–2.5)	7.3 (2.3–29.7)	2.3 (0.9–3.95)	45.77	**<0.001**	Pos > Neg (**p < 0.001**); Pos > Susp (**p = 0.011**); Neg vs Susp ns (p = 0.701)
CA 125	10.0 (7.0–19.5)	32.6 (16.0–55.0)	21.0 (8.0–29.0)	13.43	**0.001**	Pos > Neg (**p < 0.001**); Pos vs Susp ns (p = 1); Neg vs Susp ns (p = 0.319)

[¹⁸F]NaF: sodium fluoride (¹⁸F); PET: positron emission tomography; IQR: interquartile range; CA 15-3: cancer antigen 15-3; CEA: carcinoembryonic antigen; CA 125: cancer antigen 125; χ²: chi-square; Pos: positive; Neg: negative; Susp: suspicious (equivocal); ns: not significant. P-values < 0.05 were considered statistically significant. Bold values indicate statistically significant results.

### 3.3 Association between lesion count and tumor marker levels

A Kruskal–Wallis test showed significant differences across lesion-count categories for CA 15-3 (H = 83.210, df = 6, p < 0.001), CEA (H = 57.752, df = 6, p < 0.001), and CA 125 (H = 20.567, df = 6, p = 0.002). *Post-hoc* pairwise Mann–Whitney U tests (Bonferroni-corrected) demonstrated a statistically significant association between lesion count and tumor marker levels. Specifically, patients with >5 lesions had significantly higher CA 15–3 than those with 0 (U = 17112.5, Z = −8.279, p < 0.001), 1 (U = 420.5, Z = −3.488, p < 0.001), 2 (U = 319, Z = −2.936, p = 0.003), and 4 lesions (U = 108, Z = −2.991, p = 0.003). For CEA, levels were significantly elevated in >5 versus 0 lesions (U = 8862, Z = −7.281, p < 0.001), 3 lesions (U = 28.5, Z = −2.511, p = 0.012), and 5 lesions (U = 40, Z = −2.042, p = 0.041). CA 125 was significantly higher in >5 versus 0 lesions (U = 12057.5, Z = −3.332, p = 0.001) and versus 3 lesions (U = 58, Z = −1.973, p = 0.049). Comprehensive comparisons can be found in the **Supplementary Table** included in the **Supplementary Materials**. The relationship between lesion count and marker levels was significant overall, with markedly elevated CA 15-3, CEA, and CA 125 levels in patients with more than five lesions compared with those without lesions.

**Figure 1:**
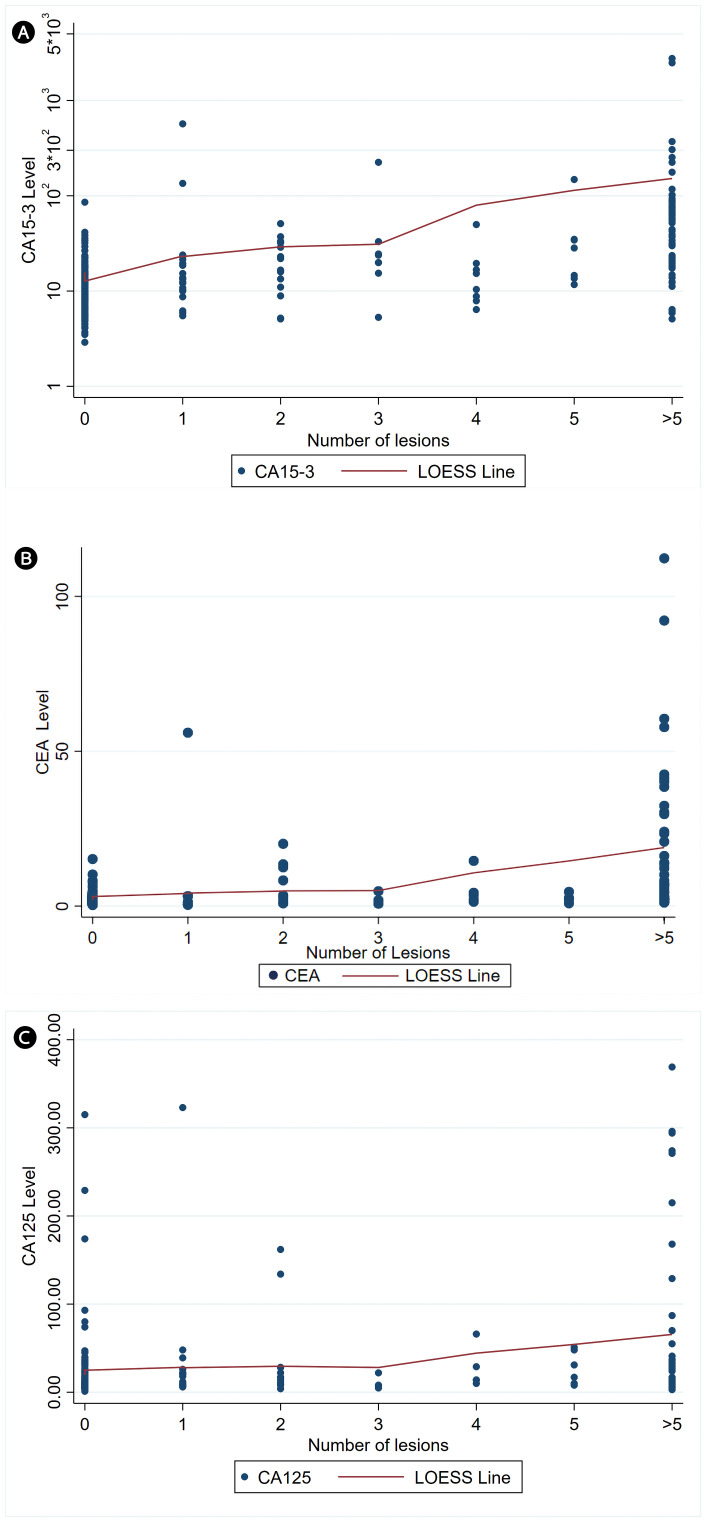
Association between skeletal lesion count on [^18^F]NaF PET and serum levels of tumor markers: **(A)** CA 15-3, **(B)** CEA, and **(C)** CA 125. Linear regression on log-transformed tumor markers showed significant positive associations with lesion count for **(A)** CA 15-3 (B: 0.23; 95% CI: 0.19–0.27; p < 0.001), corresponding to a 26% increase per additional lesion category (95% CI, 21–31%), and for **(B)** CEA (B: 0.28; 95% CI: 0.22–0.34; p < 0.001), equivalent to a 32% increase per lesion category (95% CI, 24–40%). For **(C)** CA 125, regression assumptions were not fully met; Spearman correlations showed a weaker but significant positive association with lesion count (ρ = 0.26, p < 0.001). Locally Estimated Scatterplot Smoothing (LOESS) curves are overlaid to illustrate observed trends. [^18^F]NaF PET: Sodium [^18^F]fluoride positron emission tomography; CA: cancer antigen; CEA: carcinoembryonic antigen.

To assess whether this relationship reflected a continuous dose–response effect rather than only threshold differences at high metastatic burden, we performed trend analyses. In linear regression models with log-transformed tumor markers, the number of lesions was significantly associated with higher CA 15–3 levels (B: 0.23, 95% CI: 0.19–0.27, p < 0.001), corresponding to an estimated 26% increase per additional lesion category (95% CI: 21–31%). The model explained 32% of the variance in CA 15-3 (R² = 0.32). Similarly, CEA showed a significant positive association with lesion count (B: 0.28, 95% CI: 0.22–0.34, p < 0.001), equivalent to a 32% increase per additional lesion category (95% CI: 24–40%), with the model accounting for 30% of the variance (R² = 0.30). For CA 125, regression diagnostics suggested that log transformation did not fully meet model assumptions, and the explained variance was modest (R² ≈ 0.07). Accordingly, a non-parametric Spearman correlation was used, revealing a weaker but significant positive correlation with lesion count (ρ = 0.26, p < 0.001). LOESS curves are overlaid on the scatter plots in **Figure 1** for each marker (A: CA 15-3; B: CEA; C: CA 125) to illustrate observed trends. Together, these analyses demonstrate both threshold effects at high metastatic burden and consistent upward trends across the spectrum of lesion counts.

### 3.4 Logistic regression analysis of predictors of [^18^F]NaF PET positivity

Logistic regression analysis, adjusted for age, CA 15-3, CA 125, CEA, ALP, and LDH, was performed to identify factors associated with [^18^F]NaF PET positivity. Among the included variables, CA 15–3 and CEA were significantly associated with PET positivity. Each unit increase in CA 15–3 was associated with a 5.3% higher odds (OR 1.053, 95% CI 1.020–1.087, P = 0.002), while each unit increase in CEA increased the odds by 26.4% (OR 1.264, 95% CI 1.099–1.455, P = 0.001). CA 125 showed a marginal trend toward association (OR 1.008, 95% CI 0.999–1.018, P = 0.081). Detailed results are presented in **Table 3**. Moreover, a separate logistic regression analysis of clinicopathologic factors revealed that restaging indication (vs. staging, P < 0.001) and lobular histology (vs. ductal, P = 0.008) were significant predictors of PET positivity. Other factors, including tumor grade, ER/PR status, HER2 status, and TNM stage, were not significantly associated with PET results (**Table 4**).

**Table 3:** Logistic regression analysis of factors associated with [^18^F]NaF PET positivity.

Variable	Coefficient	SE	OR	95% CI for OR	P-value
Age	-0.003	0.018	0.997	0.962 – 1.032	0.858
CA 15-3	0.051	0.016	1.053	1.020 – 1.087	**0.002**
CA 125	0.008	0.005	1.008	0.999 – 1.018	0.081
CEA	0.234	0.072	1.264	1.099 – 1.455	**0.001**
LDH	0.002	0.005	1.002	0.992 – 1.012	0.669
ALP	0.007	0.008	1.007	0.991 – 1.024	0.402

[¹⁸F]NaF: sodium fluoride (¹⁸F); SE: standard error; OR: odds ratio; CI: confidence interval; CA 15-3: cancer antigen 15-3; CA 125: cancer antigen 125; CEA: carcinoembryonic antigen; LDH: lactate dehydrogenase; ALP: alkaline phosphatase. P-values < 0.05 were considered statistically significant. Bold values indicate statistically significant results.

**Table 4:** Logistic regression analysis for [^18^F]NaF PET results.

Variable	Coefficient	SE	z-value	OR	95% CI (OR)	P-value
HER2 Status (Positive)	0.14	0.58	0.24	1.15	0.37 – 3.60	0.81
ER Status (Positive)	1.01	0.90	1.12	2.75	0.47 – 16.12	0.26
PR Status (Positive)	-0.31	0.62	-0.50	0.73	0.21 – 2.48	0.62
Indication (Restaging)	3.18	0.78	4.14	24.05	5.31 – 107.77	**<0.001**
Histology (Lobular)	1.56	0.59	2.66	4.76	1.51 – 15.03	**0.008**
Histology (Mixed)	1.01	1.44	0.70	2.74	0.16 – 46.52	0.48
Tumor Grade (Moderately differentiated)	-0.09	0.58	-0.15	0.91	0.29 – 2.89	0.88
Tumor Grade (Poorly differentiated)	-0.86	0.77	-1.12	0.42	0.09 – 1.90	0.26
T Staging (T1)	-0.74	0.66	-1.12	0.48	0.13 – 1.73	0.12
T Staging (T2)	1.60	1.44	1.11	4.95	0.30 – 83.64	0.20
T Staging (T3)	1.42	0.65	2.17	4.14	1.15 – 14.87	0.15
T Staging (T4)	2.05	0.37	5.59	7.78	3.78 – 15.95	0.18
N Staging (N1)	0.12	0.74	0.16	1.13	0.26 – 4.79	0.30
N Staging (N2)	0.67	0.29	2.32	1.95	1.11 – 3.46	0.25
N Staging (N3)	1.00	0.91	1.10	2.72	0.46 – 16.10	0.22
M Staging (M1)	1.87	0.82	2.27	6.49	1.75 – 23.97	0.28

[¹⁸F]NaF: sodium fluoride (¹⁸F); SE: standard error; OR: odds ratio; CI: confidence interval; ER: estrogen receptor; PR: progesterone receptor; HER2: human epidermal growth factor receptor 2. P-values < 0.05 were considered statistically significant. Bold values indicate statistically significant results.

### 3.5 Diagnostic performance of CA 15-3, CEA, and CA 125

**Figure 2:**
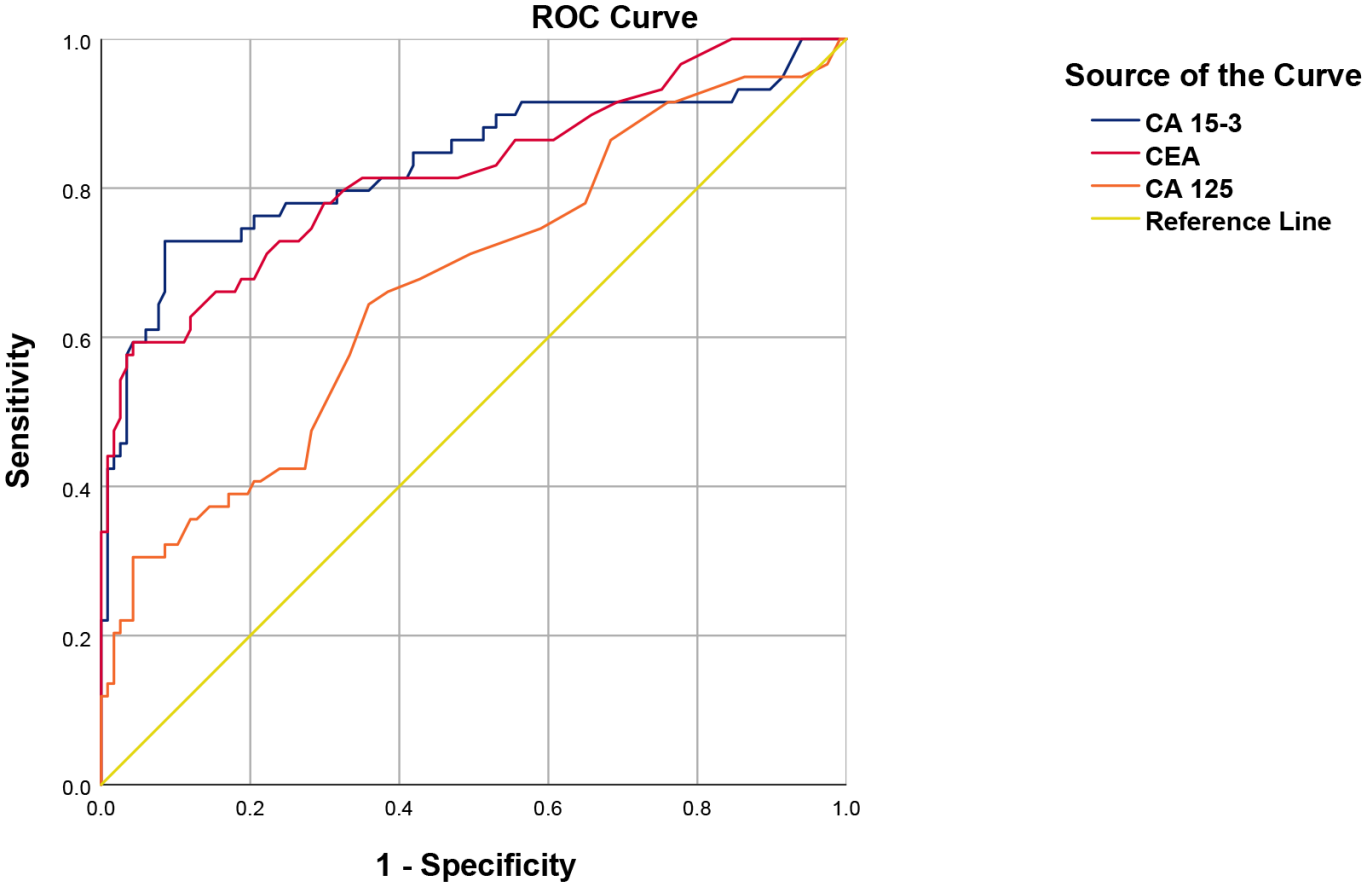
ROC analysis of CA 15-3, CEA, and CA 125 for [^18^F]NaF PET positivity. Receiver operating characteristic (ROC) curves for each tumor marker were generated to evaluate their ability to discriminate between PET-positive and PET-negative cases. Optimal cut-off values were determined using the Youden index. For CA 15-3, a threshold of 19.25 U/mL achieved 70.1% sensitivity and 90.4% specificity, with a positive predictive value (PPV) of 78.2%, negative predictive value (NPV) of 86.1%, positive likelihood ratio (LR+) of 7.38, negative likelihood ratio (LR–) of 0.33, and an AUC of 0.837 (95% CI: 0.780–0.895). For CEA, the optimal cut-off of 3.15 ng/mL yielded 65.6% sensitivity, 85.2% specificity, 67.8% PPV, 83.8% NPV, LR + 4.43, LR– 0.40, and AUC 0.821 (95% CI: 0.752–0.890). For CA 125, the cut-off of 13.5 U/mL provided 65.6% sensitivity, 62.9% specificity, 44.2% PPV, 80.4% NPV, LR + 1.77, LR– 0.55, and AUC 0.664 (95% CI: 0.582–0.746). [^18^F]NaF PET: Sodium [^18^F]fluoride positron emission tomography; CA: cancer antigen; CEA: carcinoembryonic antigen.

To evaluate the clinical utility of these markers, the diagnostic performance of individual tumor markers in predicting [^18^F]NaF PET positivity was first assessed using receiver operating characteristic (ROC) curve analysis, with optimal cut-offs determined by the Youden index. For CA 15-3, a threshold of 19.25 U/mL yielded 70.1% sensitivity, 90.4% specificity, a positive predictive value (PPV) of 78.2%, a negative predictive value (NPV) of 86.1%, LR+ of 7.38, LR– of 0.33, and an area under the curve (AUC) of 0.837 (95% CI, 0.780–0.895). CEA, at a cut-off of 3.15 ng/mL, demonstrated 65.6% sensitivity, 85.2% specificity, PPV of 67.8%, NPV of 83.8%, LR+ of 4.43, LR– of 0.40, and an AUC of 0.821 (95% CI, 0.752–0.890). CA 125, with a threshold of 13.5 U/mL, showed lower performance, with 65.6% sensitivity, 62.9% specificity, PPV of 44.2%, NPV of 80.4%, LR+ of 1.77, LR– of 0.55, and an AUC of 0.664 (95% CI, 0.582–0.746; **Figure 2**).

A multivariable logistic regression model incorporating CA 15-3, CEA, and CA 125, selected via stepwise procedures, with ALP, LDH, and age excluded, demonstrated improved discrimination for predicting [^18^F]NaF PET positivity. At a probability cut-off of 0.29 (determined by the Youden index), the model achieved a sensitivity of 79.7%, specificity of 92.3%, PPV of 83.9%, NPV of 90.0%, LR+ of 10.4, LR– of 0.22, and an AUC of 0.863 (95% CI, 0.794–0.932) (**Table 5**).

**Table 5:** Diagnostic performance of individual tumor markers and the multivariable model for predicting NAF PET positivity.

Test/Marker	Optimal cut-off (U/mL)	Sensitivity %	Specificity %	PPV %	NPV %	LR+	LR–	AUC (95% CI)
CA 15-3	19.25	70.1	90.4	78.2	86.1	7.38	0.33	0.837 (0.780–0.895)
CEA	3.15	65.6	85.2	67.8	83.8	4.43	0.40	0.821 (0.752–0.890)
CA 125	13.5	65.6	62.9	44.2	80.4	1.77	0.55	0.664 (0.582–0.746)
Final model^*^	0.29^§^	79.7	92.3	83.9	90	10.4	0.22	0.863 (0.794-0.932)

^*^Final multivariable logistic regression model incorporating CA 15-3, CEA, and CA 125, selected via stepwise procedures (ALP, LDH, and age excluded), with a probability cut-off of 0.29. **^§^**Probability cut-off. PPV, positive predictive Value; NPV, negative predictive value; LR+, positive likelihood ratio; LR–, negative likelihood ratio; AUC, area under the curve; CI, confidence interval; CA 125, Cancer antigen 125; CA 15-3, Cancer antigen 15-3; CEA, Carcinoembryonic antigen.

## 4 Discussion

To the best of our knowledge, this is the first original study to comprehensively evaluate CA 15-3, CEA, and CA 125 tumor markers as predictors of [^18^F]NaF PET positivity while adjusting for routine clinical covariates (age, ALP, and LDH). We found that levels of CA 15-3, CEA, and CA 125 were progressively elevated across categories of negative, equivocal, and positive PET scans, with significant differences between positive and negative groups for all three markers (all p < 0.001). Furthermore, a clear dose–response trend was observed between all three markers and lesion count, most pronounced for CA 15–3 and CEA, while CA 125 showed a statistically significant but comparatively weaker association, supporting their potential utility in estimating metastatic burden and guiding risk stratification. Additionally, multivariable analysis adjusted for age, ALP, and LDH confirmed that CA 15–3 and CEA were independent predictors of [^18^F]NaF PET positivity (p = 0.002 and p = 0.001, respectively), whereas CA 125 showed only a marginal trend (p = 0.081). These results indicate that CA 15–3 and CEA are independently associated with PET-positive bone metastases, reinforcing their clinical value in identifying [^18^F]NaF PET positivity. Importantly, tumor markers did not differentiate true bone metastases from benign findings in patients with initially equivocal PET scans, indicating their limited standalone value in these patients and highlighting the potential utility of threshold-based interpretation to improve diagnostic clarity. ROC analysis identified optimal cut-off values (Youden index) for predicting PET positivity, with 19.25 U/mL for CA 15-3 (sensitivity 70.1%, specificity 90.4%, LR + 7.38, AUC 0.837), 3.15 ng/mL for CEA (sensitivity 65.6%, specificity 85.2%, LR + 4.43, AUC 0.821), and 13.5 U/mL for CA 125 (sensitivity 65.6%, specificity 62.9%, LR + 1.77, AUC 0.664), indicating that CA 15–3 and CEA in particular may serve as complementary tools for PET imaging and assessing skeletal metastatic burden in breast cancer. Subsequently, a multivariable model combining CA 15-3, CEA, and CA 125, selected through a stepwise process, further improved diagnostic performance at a probability cut-off of 0.29, achieving a sensitivity of 79.7%, specificity of 92.3%, PPV of 83.9%, NPV of 90.0%, LR+ of 10.4, and an AUC of 0.863. These results suggest that integrating multiple tumor markers may provide a more robust tool for predicting [^18^F]NaF PET positivity and estimating skeletal metastatic burden in breast cancer patients. Moreover, multivariable clinicopathological analysis identified restaging indication and invasive lobular carcinoma histology as significant independent predictors of a positive PET scan, emphasizing the value of tailoring imaging strategies to individual patient profiles.

Our findings contribute to a growing body of evidence supporting the role of serum biomarkers as valuable adjuncts to advanced imaging in oncology. Precise detection of bone metastases is crucial for initial staging, treatment planning, monitoring therapeutic response, and identifying patients at risk for skeletal-related complications across various malignancies. While bone scintigraphy with technetium-99m–labeled diphosphonates (e.g., [^99m^Tc]Tc-MDP/HDP/DPD) has long been a cost-effective, widely used standard for whole-body skeletal surveying, often supplemented by regional SPECT or SPECT/CT, the limited spatial resolution of planar imaging and SPECT reduces sensitivity for detecting metastatic bone lesions (11, 20). The transition to higher-resolution [^18^F]NaF PET/CT has proven advantageous, particularly for assessing the extent of skeletal metastases compared with traditional imaging modalities such as [^99m^Tc]Tc-MDP bone scans in various malignancies (21–29). Several studies have demonstrated that [^18^F]NaF PET (or PET/CT) provides significantly higher sensitivity, negative predictive value, and accuracy compared to [^99m^Tc]Tc-MDP SPECT for detecting bone metastases, while also identifying a greater number of lesions in lesion-based analyses (30). These results are supported by a meta-analysis demonstrating that [^18^F]NaF PET/CT surpassed ^99m^Tc-bone scans, with or without SPECT, showing pooled patient-based sensitivity and specificity of 96.2% and 98.7%, and pooled lesion-based sensitivity and specificity of 96.9% and 98.0% (31), thereby establishing [^18^F]NaF PET/CT as a markedly superior modality for assessing the extent of skeletal metastases (32). Our study adds to these growing evidence by showing that tumor markers CA 15-3, CEA, and CA 125 correlate with [^18^F]NaF PET findings, with significantly higher marker levels in PET-positive versus PET-negative groups, a dose–response relationship between marker levels and lesion count (stronger for CA 15–3 and CEA), and confirming CA 15–3 and CEA as independent predictors of PET positivity, while CA 125 showed only a marginal trend. Prior studies have highlighted the important role of tumor markers CA 15-3, CEA, and CA 125 in enhancing the diagnostic performance of PET imaging in breast cancer (32–34). Integration of these markers with PET tracers, such as [^18^F]FDG, has been associated with improved detection of metastases and monitoring of disease progression. CEA positivity has been shown to correlate with higher metabolic tumor volume (MTV) and total lesion glycolysis (TLG) on [^18^F]FDG PET/CT, reflecting greater primary tumor burden (35). Furthermore, studies have revealed systematically higher levels of CA 15-3, CEA, and CA 125 in patients with positive [^18^F]FDG PET/CT scans (33, 34), supporting their utility in detecting recurrent or metastatic breast cancer. Longitudinal trends in CA 15–3 levels have also been shown to significantly correlate with PET/CT findings during post-therapy surveillance (35), and combining CA 15–3 with [^18^F]NaF PET has been reported to further enhance the detection of bone metastases, as higher marker levels have been associated with an increased number of lesions identified on imaging (36). Notably, our study established optimal cut-off values for CA 15-3 (19.25 U/mL), CEA (3.15 ng/mL), and CA 125 (13.5 U/mL) associated with [^18^F]NaF PET positivity, with CA 15–3 and CEA demonstrating the strongest diagnostic performance. Additionally, the introduced multivariable model combining all three markers further improved performance (LR + 10.4), highlighting their value as complementary tools to PET imaging for assessing skeletal metastatic burden in breast cancer. In this context, previous studies have shown that elevated CA 15–3 and CEA levels can aid in detecting [^18^F]FDG PET positivity in breast cancer patients (37–39). While some reported that CA 15–3 values above 60 U/mL and below 50 U/mL consistently corresponded to positive and negative [^18^F]FDG PET findings, respectively (39), others identified an optimal CA 15–3 cut-off of 19.1 U/mL (37), closely aligning with our results. Similarly, prior work reported that a CEA threshold of 4.8 ng/mL predicted confirmed tumor recurrence, albeit with limited sensitivity (33). Notably, [^18^F]FDG PET/CT was found to be more sensitive than CT and CA 15–3 for predicting relapse and remained an independent predictor on multivariable analysis, reinforcing the primacy of PET while highlighting the adjunctive value of tumor markers for patient triage and risk stratification (37). Collectively, these findings support integrating tumor marker assessment with PET imaging to optimize evaluation of skeletal metastatic burden and guide clinical decision-making. Consistently, our study found that CA 15–3 and CEA were significant predictors of [^18^F]NaF PET positivity, and a separate logistic regression further identified restaging referrals and lobular histology as additional independent predictors of [^18^F]NaF PET positivity, underscoring the importance of integrating tumor biology and clinical context into imaging decisions to optimize patient management.

The findings of this study have direct implications for optimizing the diagnostic pathway for breast cancer patients at risk of bone metastases. Our data support a strategic, risk-adapted approach to employing [^18^F]NaF PET, guided by serum tumor marker levels. The primary clinical utility of CA 15–3 and CEA lies in rule-in and risk-stratification strategies, as demonstrated by their high specificity (CA 15-3: 90.4%; CEA: 85.2%) and moderate-to-strong positive likelihood ratios (CA 15-3: LR + 7.38; CEA: LR + 4.43) at the identified cut-offs, a performance that was further improved in a combined model of all three markers (LR + 10.4). Therefore, patients exceeding these thresholds, particularly those with additional risk factors such as invasive lobular carcinoma or a restaging indication, should be prioritized for [^18^F]NaF PET evaluation. This can help reduce waiting times for high-risk patients and ensure timely treatment initiation. Notably, the CA 15–3 threshold of 19.25 U/mL is lower than conventional laboratory reference limits. This is a critical finding, as it indicates that PET-detectable metastases can occur even when CA 15–3 levels are within the “normal” range by routine laboratory reference standards. Clinicians should therefore not rely solely on standard reference ranges to defer imaging in patients with a high clinical suspicion of metastatic disease. Instead, this lower, PET-optimized threshold can guide more aggressive screening and early PET prioritization. Furthermore, in cases of equivocal PET findings, elevated marker levels can enhance interpretive confidence or guide planning for a shorter follow-up interval. Nevertheless, it is crucial to emphasize that the modest sensitivity of these markers (~ 65-70%) precludes their use for ruling out disease. Patients with marker levels below these cut-offs cannot be safely excluded from imaging and should continue on standard surveillance schedules. In this context, the markers function not as a gatekeeper to prevent imaging, but as a triage tool to identify who needs it most urgently. In summary, integrating tumor markers, particularly CA 15–3 and CEA, into clinical decision-making enables a more nuanced, risk-adapted approach. By using these biomarkers to identify patients with a high pre-test probability of bone metastases, clinicians can optimize resource allocation, improve the timeliness of metastasis detection, and enhance the interpretation of complex imaging findings.

Several limitations should be acknowledged. While this study highlights the link between serum tumor markers and [^18^F]NaF PET findings, its retrospective, single-center design, lack of biopsy confirmation for all patients (reflecting routine clinical practice), modest marker sensitivity, and absence of external validation are important considerations. Although PET was the primary modality, CT was used selectively, and semiquantitative metrics (SUVmax, total lesion activity, MTV) were unavailable, reflecting current clinical practice, where their reproducibility and added value remain limited. The retrospective design also precluded assessment of biomarker trends and full control for potential confounders (e.g., inflammation, hepatic dysfunction, or benign conditions), while unblinded PET interpretation relative to clinical data could introduce bias. Limited lobular carcinoma cases and incomplete tumor marker data constrained stratified analyses by histology, whereas receptor status (ER, PR, HER2) and tumor grade were not significant predictors of [^18^F]NaF PET positivity, likely reflecting small subgroups and absent molecular subtype stratification. Future prospective, multi-center, subtype-specific studies with larger cohorts and stricter biomarker–imaging alignment are needed to validate cut-offs, clarify subtype effects, and confirm clinical utility.

The promising associations identified here provide a clear roadmap for future research aimed at translating diagnostic findings into clinical impact. Critical next steps include: (1) establishing prognostic value by linking tumor marker levels and [^18^F]NaF PET findings to longitudinal outcomes, such as progression-free and overall survival; (2) evaluating their utility in therapy response assessment by examining whether serial measurements of CA 15–3 and CEA, together with [^18^F]NaF PET, can serve as early, sensitive biomarkers for monitoring treatment efficacy in bone-metastatic breast cancer; and (3) refining risk-adapted imaging protocols by integrating these serum markers with PET results to optimize patient triage, imaging frequency, and personalized follow-up strategies.

## 5 Conclusion

Elevated CA 15–3 and CEA independently predict [^18^F]NaF PET positivity in breast cancer. The optimal cut-offs for predicting PET positivity were 19.25 U/mL for CA 15-3 (sensitivity 70.1%, specificity 90.4%) and 3.15 ng/mL for CEA (sensitivity 65.6%, specificity 85.2%). The clinical utility of CA 15–3 and CEA lies primarily in rule-in and risk-stratification strategies. Elevated CA 15–3 and CEA demonstrated high specificity (as noted above) and moderate-to-strong positive likelihood ratios (CA 15-3: LR + 7.38; CEA: LR + 4.43), suggesting that patients above these thresholds, particularly those with invasive lobular carcinoma or undergoing restaging, may benefit from prioritized PET evaluation or improved interpretation of equivocal PET findings. Moreover, the CA 15–3 threshold identified in this study is lower than routine laboratory reference limits, suggesting that these findings may guide aggressive screening and prioritization of [^18^F]NaF PET in patients with high clinical suspicion. Notably, a multivariable model combining CA 15-3, CEA, and CA 125 further improved diagnostic performance (LR + 10.4). Despite these advantages, the modest sensitivity of these markers means that patients with lower levels cannot be safely excluded from imaging and should continue on standard surveillance schedules. Integrating CA 15–3 and CEA into clinical decision-making can therefore enable a more nuanced, risk-adapted approach, optimizing resource allocation and timeliness of metastasis detection. Prospective, multi-center, subtype-specific validation studies are warranted to confirm the clinical impact of this strategy and to further investigate its utility in prognosis and therapy response.

## Data Availability

The raw data supporting the conclusions of this article will be made available by the authors, without undue reservation.

## Glossary

MRI: Magnetic Resonance Imaging
PET: Positron Emission Tomography
CT: Computed Tomography
[^99m^Tc]Tc-MDP: Technetium-99m-labeled Methylene Diphosphonate
[^99m^Tc]Tc-HDP: Technetium-99m-labeled Hydroxymethylene Diphosphonate
[^99m^Tc]Tc-DPD: Technetium-99m-labeled 3,3-Diphosphono-1,2-Propanodicarboxylic Acid
[^18^F]NaF: Sodium Fluoride (¹⁸F)
PET/CT: Positron Emission Tomography/Computed Tomography
CA 15-3: Cancer Antigen 15-3
CEA: Carcinoembryonic Antigen
CA 125: Cancer Antigen 125
SPECT: Single-Photon Emission Computed Tomography
[^18^F]FDG: Fluorodeoxyglucose (¹⁸F)
MUHC: McGill University Health Centre
REB: Research Ethics Board
SD: Standard Deviation
IQR: Interquartile Range
H: Kruskal-Wallis H Statistic
df: Degrees of Freedom
U: Mann-Whitney U Statistic
Z: Standardized Test Statistic
B: Unstandardized Regression Coefficient
SE: Standard Error
t: t-statistic
CI: Confidence Interval
ρ (rho): Spearman’s Rank Correlation Coefficient
LOESS: Locally Estimated Scatterplot Smoothing
OR: Odds Ratio
ALP: Alkaline Phosphatase
LDH: Lactate Dehydrogenase
ROC: Receiver Operating Characteristic
PPV: Positive Predictive Value
NPV: Negative Predictive Value
LR+: Positive Likelihood Ratio
LR–: Negative Likelihood Ratio
AUC: Area Under the Curve
ER: Estrogen Receptor
PR: Progesterone Receptor
HER2: Human Epidermal Growth Factor Receptor 2
TNM: Tumor, Node, Metastasis (staging system)
ILC: Invasive Lobular Carcinoma
MTV: Metabolic Tumor Volume
TLG: Total Lesion Glycolysis
SUVmax: Maximum Standardized Uptake Value.
