# Supplementary material for: Association of tumor markers CA 15-3, CEA, and CA 125 with [18F]NaF PET findings in breast cancer patients
Source: Front Oncol. 2025 Nov 21;15:1673504. doi: 10.3389/fonc.2025.1673504 (PMC12678091; doi:10.3389/fonc.2025.1673504)
Supplement: Supplementary file 1 [file DataSheet1.zip › Supplementary Materials.DOCX]

**Supplementary Table. Comparison of tumor marker levels (CA 15-3, CEA, and CA 125) across lesion count categories**

|  | Comparison | Mean Rank (G1 / G2) | Sum of Ranks (G1 / G2) | U | W | Z | P-value  (2-tailed) |
| --- | --- | --- | --- | --- | --- | --- | --- |
| CA 15-3 | 0 vs 1 | 96.63 / 121.18 | 17200.5 / 2302.5 | 1269.5 | 17200.5 | -1.784 | 0.074 |
|  | 0 vs 2 | 93.64 / 136.90 | 16667.5 / 2053.5 | 736.5 | 16667.5 | -2.881 | **0.004** |
|  | 0 vs 3 | 90.97 / 149.69 | 16193.5 / 1197.5 | 262.5 | 16193.5 | -3.018 | **0.003** |
|  | 0 vs 4 | 92.83 / 108.31 | 16524.5 / 866.5 | 593.5 | 16524.5 | -0.796 | 0.426 |
|  | 0 vs 5 | 90.70 / 151.43 | 16145 / 1060 | 214.0 | 16145 | -2.943 | **0.003** |
|  | 0 vs >5 | 96.14 / 182.71 | 17112.5 / 9683.5 | 1181.5 | 17112.5 | -8.279 | **<0.001** |
|  | 1 vs 2 | 15.68 / 19.80 | 298 / 297 | 108.0 | 298 | -1.197 | 0.231 |
|  | 1 vs 3 | 12.32 / 18.00 | 234 / 144 | 44.0 | 234 | -1.699 | 0.089 |
|  | 1 vs 4 | 14.32 / 13.25 | 272 / 106 | 70.0 | 106 | -0.319 | 0.750 |
|  | 1 vs 5 | 11.89 / 17.86 | 226 / 125 | 36.0 | 226 | -1.763 | 0.078 |
|  | 1 vs >5 | 22.13 / 41.65 | 420.5 / 2207.5 | 230.5 | 420.5 | -3.488 | **<0.001** |
|  | 2 vs 3 | 11.60 / 12.75 | 174 / 102 | 54.0 | 174 | -0.387 | 0.698 |
|  | 2 vs 4 | 13.20 / 9.75 | 198 / 78 | 42.0 | 78 | -1.162 | 0.245 |
|  | 2 vs 5 | 10.73 / 13.14 | 161 / 92 | 41.0 | 161 | -0.811 | 0.418 |
|  | 2 vs >5 | 21.27 / 38.25 | 319 / 2027 | 199.0 | 319 | -2.936 | **0.003** |
|  | 3 vs 4 | 10.50 / 6.50 | 84 / 52 | 16.0 | 52 | -1.682 | 0.093 |
|  | 3 vs 5 | 7.75 / 8.29 | 62 / 58 | 26.0 | 62 | -0.232 | 0.817 |
|  | 3 vs >5 | 22.56 / 32.27 | 180.5 / 1710.5 | 144.5 | 180.5 | -1.442 | 0.149 |
|  | 4 vs 5 | 6.38 / 9.86 | 51 / 69 | 15.0 | 51 | -1.504 | 0.132 |
|  | 4 vs >5 | 13.50 / 33.64 | 108 / 1783 | 72.0 | 108 | -2.991 | **0.003** |
|  | 5 vs >5 | 22.43 / 31.57 | 157 / 1673 | 129.0 | 157 | -1.301 | 0.193 |
| CEA | 0 vs 1 | 69.03 / 81.27 | 8836 / 894 | 580 | 8836 | -0.968 | 0.333 |
|  | 0 vs 2 | 67.0 / 101.55 | 8575.5 / 1015.5 | 319.5 | 8575.5 | -2.634 | **0.008** |
|  | 0 vs 3 | 66.5 / 66.38 | 8512.5 / 265.5 | 255.5 | 265.5 | -0.007 | 0.995 |
|  | 0 vs 4 | 65.54 / 104.40 | 8389 / 522 | 133.0 | 8389 | -2.214 | **0.027** |
|  | 0 vs 5 | 65.80 / 89.00 | 8422 / 356 | 166.0 | 8422 | -1.196 | 0.232 |
|  | 0 vs >5 | 69.23 / 133.35 | 8862 / 5334 | 606.0 | 8862 | -7.281 | **<0.001** |
|  | 1 vs 2 | 10.18 / 11.90 | 112 / 119 | 46.0 | 112 | -0.635 | 0.526 |
|  | 1 vs 3 | 8.27 / 7.25 | 91 / 29 | 19.0 | 29 | -0.393 | 0.694 |
|  | 1 vs 4 | 7.91 / 9.80 | 87 / 49 | 21.0 | 87 | -0.739 | 0.460 |
|  | 1 vs 5 | 8.09 / 7.75 | 89 / 31 | 21.0 | 31 | -0.131 | 0.896 |
|  | 1 vs >5 | 19.86 / 27.69 | 218.5 / 1107.5 | 152.5 | 218.5 | -1.546 | 0.122 |
|  | 2 vs 3 | 8.60 / 4.75 | 86 / 19 | 9.0 | 19 | -1.557 | 0.119 |
|  | 2 vs 4 | 7.85 / 8.30 | 78.5 / 41.5 | 23.5 | 78.5 | -0.184 | 0.854 |
|  | 2 vs 5 | 8.25 / 5.63 | 82.5 / 22.5 | 12.5 | 22.5 | -1.062 | 0.288 |
|  | 2 vs >5 | 18.10 / 27.35 | 181 / 1094 | 126.0 | 181 | -1.795 | 0.073 |
|  | 3 vs 4 | 3.75 / 6.00 | 15 / 30 | 5.0 | 15 | -1.230 | 0.219 |
|  | 3 vs 5 | 3.75 / 5.25 | 15 / 21 | 5.0 | 15 | -0.871 | 0.384 |
|  | 3 vs >5 | 7.13 / 24.04 | 28.5 / 961.5 | 18.5 | 28.5 | -2.511 | **0.012** |
|  | 4 vs 5 | 5.40 / 4.50 | 27 / 18 | 8.0 | 18 | -0.490 | 0.624 |
|  | 4 vs >5 | 14.30 / 24.09 | 71.5 / 963.5 | 56.5 | 71.5 | -1.571 | 0.116 |
|  | 5 vs >5 | 10.00 / 23.75 | 40 / 950 | 30.0 | 40 | -2.042 | **0.041** |
| CA 125 | 0 vs 1 | 77.32 / 103.94 | 11057 / 1663 | 761.0 | 11057 | -2.196 | **0.028** |
|  | 0 vs 2 | 76.25 / 98.83 | 10904 / 1186 | 608.0 | 10904 | -1.676 | 0.094 |
|  | 0 vs 3 | 75.28 / 52.30 | 10764.5 / 261.5 | 246.5 | 261.5 | -1.180 | 0.238 |
|  | 0 vs 4 | 73.48 / 103.8 | 10507 / 519 | 211.0 | 10507 | -1.557 | 0.119 |
|  | 0 vs 5 | 73.77 / 104.42 | 10548.5 / 626.5 | 252.5 | 10548.5 | -1.707 | 0.088 |
|  | 0 vs >5 | 84.32 / 116.14 | 12057.5 / 4413.5 | 1761.5 | 12057.5 | -3.332 | **0.001** |
|  | 1 vs 2 | 14.81 / 14.08 | 237 / 169 | 91.0 | 169 | -0.232 | 0.816 |
|  | 1 vs 3 | 12.47 / 6.30 | 199.5 / 31.5 | 16.5 | 31.5 | -1.946 | 0.052 |
|  | 1 vs 4 | 10.84 / 11.50 | 173.5 / 57.5 | 37.5 | 173.5 | -0.207 | 0.836 |
|  | 1 vs 5 | 11.22 / 12.25 | 179.5 / 73.5 | 43.5 | 179.5 | -0.332 | 0.740 |
|  | 1 vs >5 | 26.94 / 27.74 | 431 / 1054 | 295.0 | 431 | -0.171 | 0.865 |
|  | 2 vs 3 | 10.46 / 5.50 | 125.5 / 27.5 | 12.5 | 27.5 | -1.851 | 0.064 |
|  | 2 vs 4 | 8.83 / 9.40 | 106 / 47 | 28.0 | 106 | -0.211 | 0.833 |
|  | 2 vs 5 | 9.13 / 10.25 | 109.5 / 61.5 | 31.5 | 109.5 | -0.423 | 0.673 |
|  | 2 vs >5 | 23.67 / 26.08 | 284 / 991 | 206.0 | 284 | -0.500 | 0.617 |
|  | 3 vs 4 | 3.60 / 7.40 | 18 / 37 | 3.0 | 18 | -1.997 | **0.046** |
|  | 3 vs 5 | 3.80 / 7.83 | 19 / 47 | 4.0 | 19 | -2.027 | **0.043** |
|  | 3 vs >5 | 11.60 / 23.37 | 58 / 888 | 43.0 | 58 | -1.973 | **0.049** |
|  | 4 vs 5 | 5.90 / 6.08 | 29.5 / 36.5 | 14.5 | 29.5 | -0.092 | 0.927 |
|  | 4 vs >5 | 19.90 / 22.28 | 99.5 / 846.5 | 84.5 | 99.5 | -0.398 | 0.690 |
|  | 5 vs >5 | 22.42 / 22.51 | 134.5 / 855.5 | 113.5 | 134.5 | -0.017 | 0.986 |

CA 15-3: Cancer antigen 15-3; CA 125: Cancer antigen 125; CEA: Carcinoembryonic antigen; N: Sample size; G1 / G2: Group 1 / Group 2; U: Mann–Whitney U statistic; W: Wilcoxon W statistic; Z: Standardized test statistic; Values are derived from pairwise Mann–Whitney U tests following a significant Kruskal–Wallis test. P < 0.05 was considered statistically significant.
